# Genome Sequence of the Nonconventional Wine Yeast *Hanseniaspora guilliermondii* UTAD222

**DOI:** 10.1128/genomeA.01515-16

**Published:** 2017-02-02

**Authors:** Isabel Seixas, Catarina Barbosa, Sara B. Salazar, Arlete Mendes-Faia, Yu Wang, Ulrich Güldener, Ana Mendes-Ferreira, Nuno P. Mira

**Affiliations:** a Escola de Ciências da Vida e Ambiente, Universidade de Trás-Os-Montes e Alto Douro, Vila Real, Portugal; b BioISI-Biosystems and Integrative Sciences Institute, Campo Grande, Lisbon, Portugal; c Leibniz Supercomputing Centre, Garching, Germany; d Department of Genome-oriented Bioinformatics, Wissenschaftszentrum Weihenstephan, Technische Universität München, Freising, Germany; e Institute for Bioengineering and Biosciences, Instituto Superior Técnico, Department of Bioengineering, Universidade de Lisboa, Lisbon, Portugal

## Abstract

In this work, we disclose the genome sequence and a corresponding manually curated annotation of the non-*Saccharomyces* yeast *Hanseniaspora guilliermondii* UTAD222, a strain shown to have interesting oenological traits for the production of wines with improved aromatic properties.

## GENOME ANNOUNCEMENT

Non-*Saccharomyces* yeasts have several interesting metabolic and enzymatic properties that are absent in *Saccharomyces cerevisiae* and that contribute to the improvement of wine sensory profiles (1–4). Species of the *Hanseniaspora* genus are among those found to be more interesting for the production of wines with stylistic properties in coculture with *S. cerevisiae*. In this work, we have sequenced the genome of *Hanseniaspora guilliermondii* UTAD222, a strain that was isolated from a wine must and that was found to have multiple interesting oenological traits, including high tolerance to ethanol, low production of H_2_S, and high proteolytic and β-glycosidase activities (1, 5). Coculture of *H. guilliermondii* UTAD222 with *S. cerevisiae* also significantly enhanced the floral and the fruity character of wines (6), and led to a significant remodeling of *S. cerevisiae* genomic expression along alcoholic fermentation (7). Other genomic sequences of *Hanseniaspora* wine strains were released including *H. opuntiae*, *H. uvarum*, *H. vinae*, and *H. osmophila* (BioProjects PRJNA325557, PRJNA238564, and PRJNA178141) (8), but this is the first description of a whole-genome of an *H. guilliermondii* strain.

The genome of *H. guilliermondii* UTAD222 was obtained using two rounds of paired-end sequencing using Illumina MiSeq platform (inserts with approximately 300 bp). Around 53,913,308 reads were obtained, which were assembled (using a CLC *de novo* assembler) into 208 contigs (*N*_50_ length, 91,417 bp), yielding a total of 9,037,850 assembled bases and a coverage of 819-fold. The predicted *H. guilliermondii* UTAD222 genome size and corresponding G+C content (approximately 31%) are in line with the genome sizes reported for other wine strains belonging to the *Hanseniaspora* genus.

Automatic annotation of the genome sequence of *H. guilliermondii* UTAD222 (undertaken using *ab initio* gene detection) was manually curated to validate and refine the results obtained. The predicted ORFeome of *H. guilliermondii* UTAD222 is estimated to include 4,070 protein-encoding genes. BLASTP analysis revealed that the majority of *H. guilliermondii* proteins share a high degree of similarity with proteins from *H. opuntiae* AWRI3578 (*n* = 4,026) and from *H. uvarum* AWRI3580 (*n* = 3,918), reflecting the reported close phylogenetic distance between *H. guilliermondii* and these two species (9). Despite this generalized similarity, *H. guilliermondii* UTAD222 and *H. uvarum* AWRI3580 strains were found to share three proteins that were absent in *H. opuntiae* AWRI3578. On the other hand, four genes were shared by *H. guilliermondii* UTAD222 and *H. opuntiae* AWRI3578 but were absent in *H. uvarum*. The disclosure of the *H. guilliermondii* genome sequence is expected to boost research focused on this species and on others belonging to the *Hanseniaspora* genus, helping to further understand their role in wine fermentation when cocultured with *S. cerevisiae*.

### Accession number(s).

The genome sequence of the *H. guilliermondii* UTAD222 sequence has been deposited in ENA (accession numbers FQNF01000001 to FQNF01000208).
